# Supplementary material for: Endothelial Cu Uptake Transporter CTR1 Senses Disturbed Flow to Promote Atherosclerosis through Cuproptosis
Source: bioRxiv. 2025 Jan 28:2025.01.27.634587. Preprint. [Version 1] doi: 10.1101/2025.01.27.634587 (PMC11838200; doi:10.1101/2025.01.27.634587)

bioRxiv preprint doi: https://doi.org/10.1101/2025.01.27.634587; this version posted January 28, 2025. The copyright holder for this preprint (which was not certified by peer review) is the author/funder, who has granted bioRxiv a license to display the preprint in perpetuity. It is made available under aCC-BY 4.0 International license.

Supplemental Figure Legend:

Figure S1. Effect of L-flow and D-flow on Cu uptake transporter CTR1 expression in HAECs. A and **B.** HAEC were exposed with LF (L-flow) or DF (D-flow) for indicated times. CTR1 mRNA expressions were measured using quantitative polymerase chain reaction (**A**). To examine CTR1 protein expressions, cell lysates were immunoblotted with the indicated antibodies (n=3) (**B**).

Figure S2. Ctr1 expression is increased in a DF-exposed carotid artery of *Apoe*$^{-/-}$ mice Immunofluorescence staining of Ctr1 in RCA and LCA of *Apoe*$^{-/-}$ mice 2 weeks after PCL surgery on LCA (n=3).

Figure S3. CTR1 expression in endothelial cells and characterization of inducible endothelial *Ctr1*-deficient mice. A. Strategy to generate tamoxifen-inducible EC-specific *Ctr1*-deficient (*Ctr1*^{iECKO}) mice on *Apoe*$^{-/-}$ background by crossing *Ctr1*$^{\text{flox/flox}}$/*Apoe*$^{-/-}$ mice with *Cdh5-CreER*^{T2}/*Apoe*$^{-/-}$ mice. **B** and **C.** Ctr1 mRNA expression was measured by quantitative polymerase chain reaction in EC or aorta or liver of *Ctr1*$^{\text{flox/flox}}$/*Apoe*$^{-/-}$ mice (**B**) and CTR1 siRNA transfected HAEC cells (**C**) n=4.

Figure S4. Lipid levels were not changed in TTM treated, EC-Specific *Ctr1*-deficient or mitoCDN-treated *Apoe*$^{-/-}$ mice fed with western diet. Plasma total, LDL or HDL cholesterol and triglyceride levels were measured in mice after overnight fasting in *Ctr1*^{iECKO}/*Apoe*$^{-/-}$ (A), TTM treated (B), mitoCDN treated (C) *Apoe*$^{-/-}$ mice (n=6-7).

Figure S5. TTM treatment decreased plasma ceruloplasmin activity. Treatment and surgery protocol for these mice are shown in Fig. 1G. **(A)** *Apoe*$^{-/-}$ mice treated with Cu chelator tetrathiomolybdate (TTM) and western diet (WD) received partial carotid ligation (PCL) surgery (3 weeks) on left carotid artery (LCA), n=8. Plasma ceruloplasmin activity was measured.

Figure S6. Iron or Zinc contents were not changed in cytosolic or mitochondrial fractions of DF exposed HAECs. After flow exposure in HAECs for 4 or 24 hrs, Fe and Zn levels were measured in cytosolic and mitochondrial fractions of cells (n=3-4) using ICP-MS.

Figure S7. Role of mitochondrial Cu in DF-induced decrease in the protein expression of oxidative phosphorylation complex proteins. (A-C) HAEC were transfected with control siRNA or CTR1 siRNA (**A**) or TTM (20nM, 24 hrs) (**B**) or mitoCDN (1 µM, 1 hr) (**C**) and exposed with flow for 48 hrs. Cell lysate were immunoblotted with the indicated antibodies (n=3-4).

bioRxiv preprint doi: https://doi.org/10.1101/2025.01.27.634587; this version posted January 28, 2025. The copyright holder for this preprint (which was not certified by peer review) is the author/funder, who has granted bioRxiv a license to display the preprint in perpetuity. It is made available under aCC-BY 4.0 International license.

Figure S8. Cu chelator TTM or CTR1 siRNA restored DF-induced decrease in cell viability of ECs. HAEC were transfected with control siRNA or CTR1 siRNA (24 hrs) or pretreated for 1 hr with 20 μM necrostatin-1 (Nec), 10 μM ferrostatin-1 (Fer), 30 μM Z-VAD-FMK (Apo) or pretreated with 20 nM TTM for 24 hrs. The cells were then exposed flow for 48 hrs. Cell death was measured by CCK8 assay (n=4) or LDH release from conditioned medium (n=4).

Figure S9. Mitochondrial-specific Cu chelator mitoCDN prevented DF-induced mitochondrial dysfunction *ex vivo*. A, Schematic diagram of experimental design for Figure 3G and S9B **B**, *Apoe*$^{-/-}$ mice were treated with mitoCDN (1.35mg/kg) every 3 days with total of 10 doses and fed western diet for 30 days. Mitochondrial respiratory capacity measured in endothelial cells of aortic arch (D-flow, DF) and thoracic aorta (L-flow, LF) of aorta by O_2 consumption rate (OCR) using a Seahorse analyzer (n=5).

Figure S10. Knockdown of SLC25A3 or CTR1 did not change Fe or Zn levels in flow-exposed ECs. HAECs were transfected with control siRNA or SLC25A3 siRNA or CTR1 siRNA (48 hrs) and exposed to flow for 24 hrs. Iron (Fe) or Zinc (Zn) levels were measured by ICP-MS in cytosolic and mitochondrial fraction of cells (n=3-4).

Figure S11. Knockdown of SLC25A3 or Cav1 restored DF-induced decrease in cuproptosis-related protein expression in ECs. (A and B) HAECs were transfected with SLC25A3 siRNA (A) or Cav1 siRNA (B) or control and exposed flow for 48 hrs and cell lysates were immunoblotted with the indicated antibodies (n=3-4).

bioRxiv preprint doi: https://doi.org/10.1101/2025.01.27.634587; this version posted January 28, 2025. The copyright holder for this preprint (which was not certified by peer review) is the author/funder, who has granted bioRxiv a license to display the preprint in perpetuity. It is made available under aCC-BY 4.0 International license.

Supplementary Figure 1

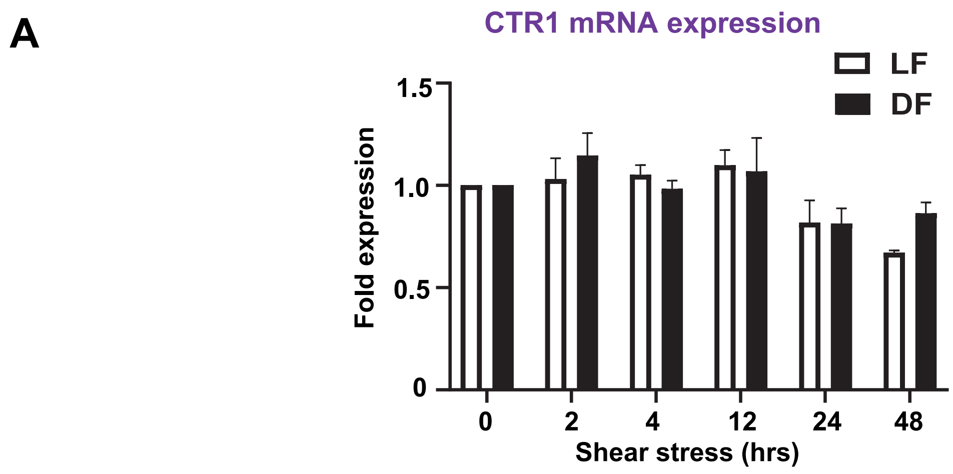

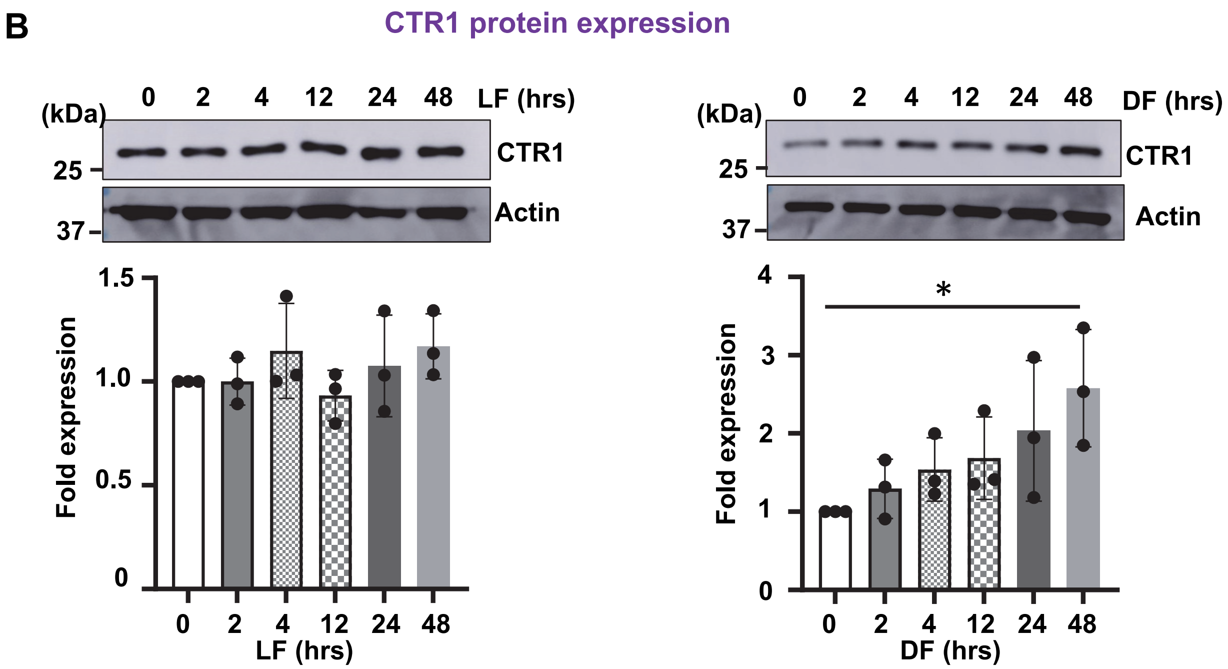

bioRxiv preprint doi: https://doi.org/10.1101/2025.01.27.634587; this version posted January 28, 2025. The copyright holder for this preprint (which was not certified by peer review) is the author/funder, who has granted bioRxiv a license to display the preprint in perpetuity. It is made available under aCC-BY 4.0 International license.

Supplementary Figure 2

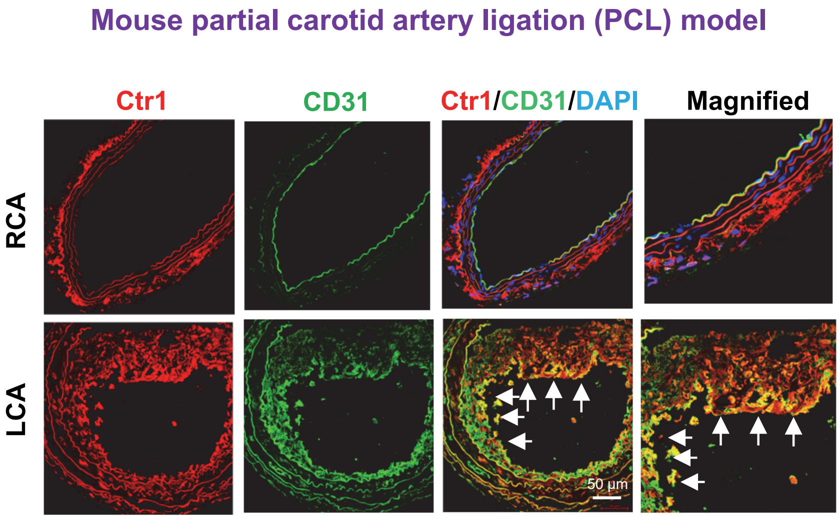

bioRxiv preprint doi: https://doi.org/10.1101/2025.01.27.634587; this version posted January 28, 2025. The copyright holder for this preprint (which was not certified by peer review) is the author/funder, who has granted bioRxiv a license to display the preprint in perpetuity. It is made available under aCC-BY 4.0 International license.

Supplementary Figure 3

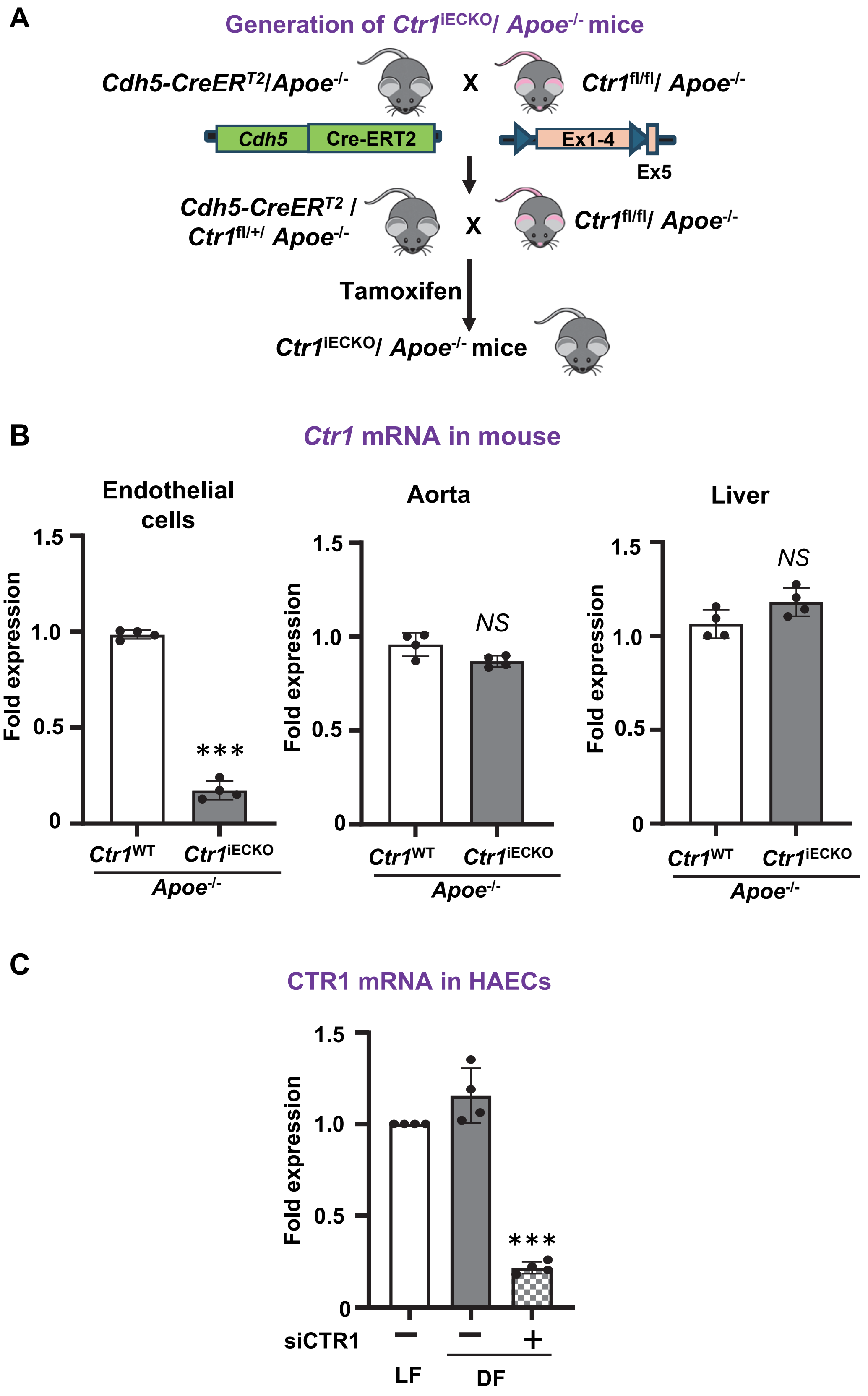

bioRxiv preprint doi: https://doi.org/10.1101/2025.01.27.634587; this version posted January 28, 2025. The copyright holder for this preprint (which was not certified by peer review) is the author/funder, who has granted bioRxiv a license to display the preprint in perpetuity. It is made available under aCC-BY 4.0 International license.

Supplementary Figure 4

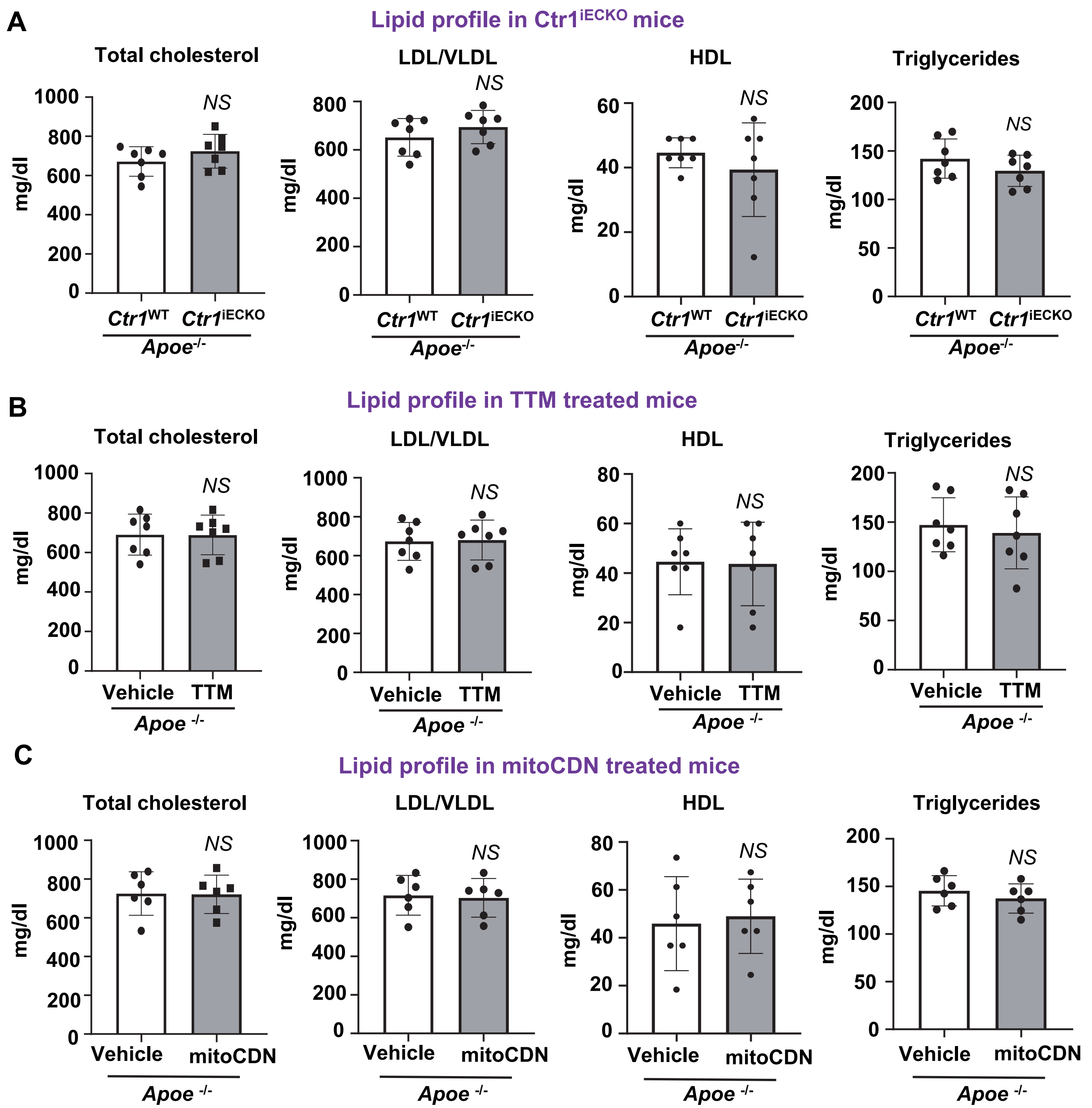

bioRxiv preprint doi: https://doi.org/10.1101/2025.01.27.634587; this version posted January 28, 2025. The copyright holder for this preprint (which was not certified by peer review) is the author/funder, who has granted bioRxiv a license to display the preprint in perpetuity. It is made available under aCC-BY 4.0 International license.

Supplementary Figure 5

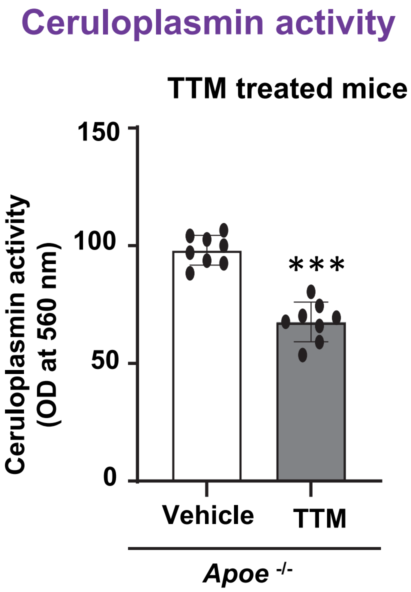

bioRxiv preprint doi: https://doi.org/10.1101/2025.01.27.634587; this version posted January 28, 2025. The copyright holder for this preprint (which was not certified by peer review) is the author/funder, who has granted bioRxiv a license to display the preprint in perpetuity. It is made available under aCC-BY 4.0 International license.

Supplementary Figure 6

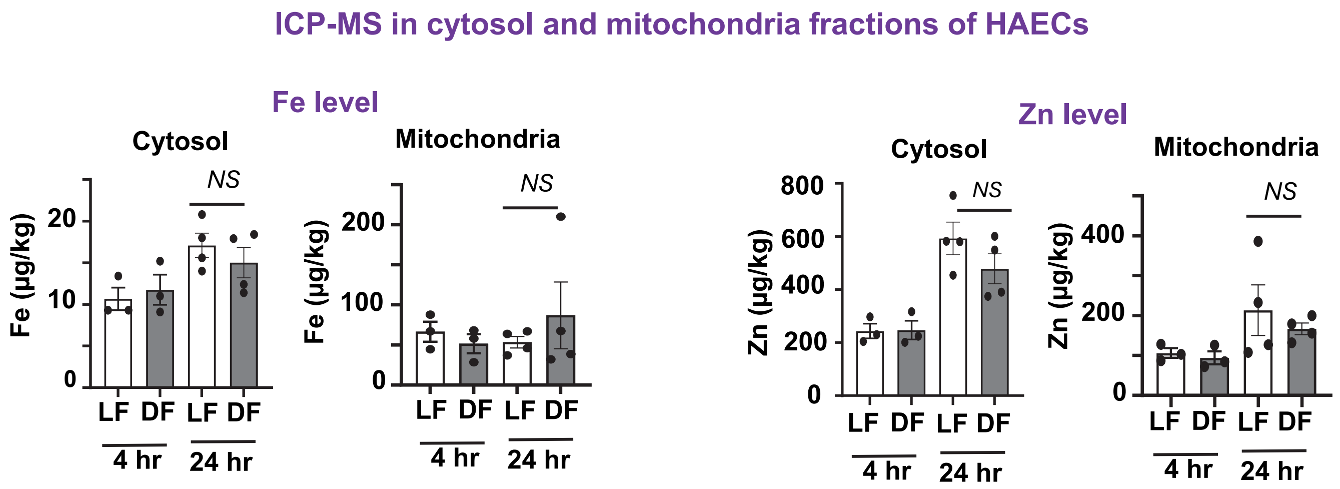

bioRxiv preprint doi: https://doi.org/10.1101/2025.01.27.634587; this version posted January 28, 2025. The copyright holder for this preprint (which was not certified by peer review) is the author/funder, who has granted bioRxiv a license to display the preprint in perpetuity. It is made available under aCC-BY 4.0 International license.

Supplementary Figure 7

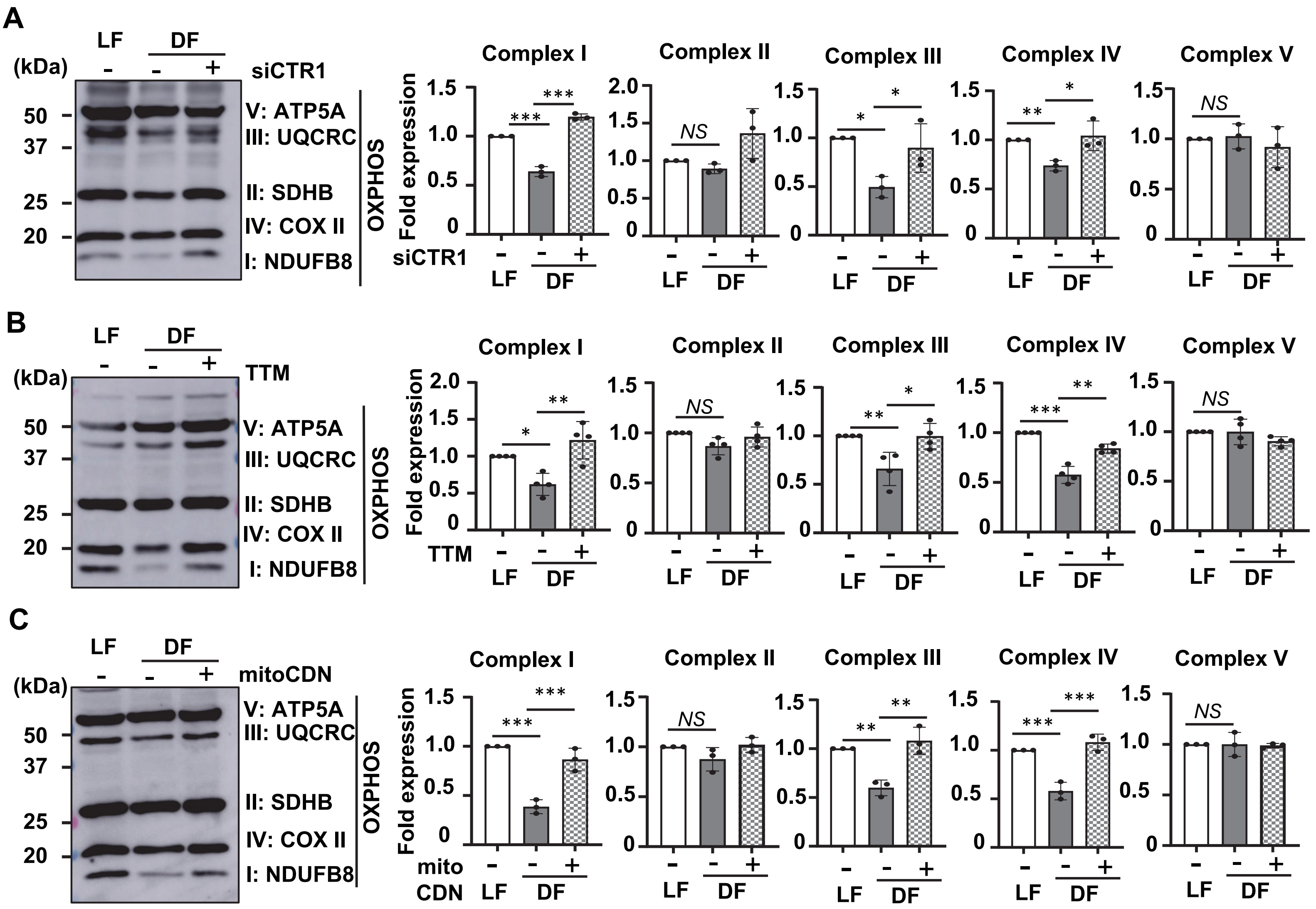

bioRxiv preprint doi: https://doi.org/10.1101/2025.01.27.634587; this version posted January 28, 2025. The copyright holder for this preprint (which was not certified by peer review) is the author/funder, who has granted bioRxiv a license to display the preprint in perpetuity. It is made available under aCC-BY 4.0 International license.

Supplementary Figure 8

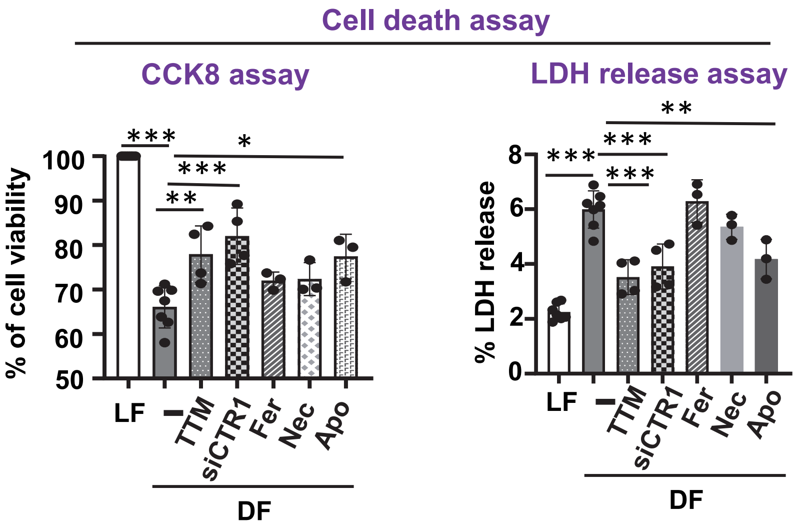

bioRxiv preprint doi: https://doi.org/10.1101/2025.01.27.634587; this version posted January 28, 2025. The copyright holder for this preprint (which was not certified by peer review) is the author/funder, who has granted bioRxiv a license to display the preprint in perpetuity. It is made available under aCC-BY 4.0 International license.

Supplementary Figure 9

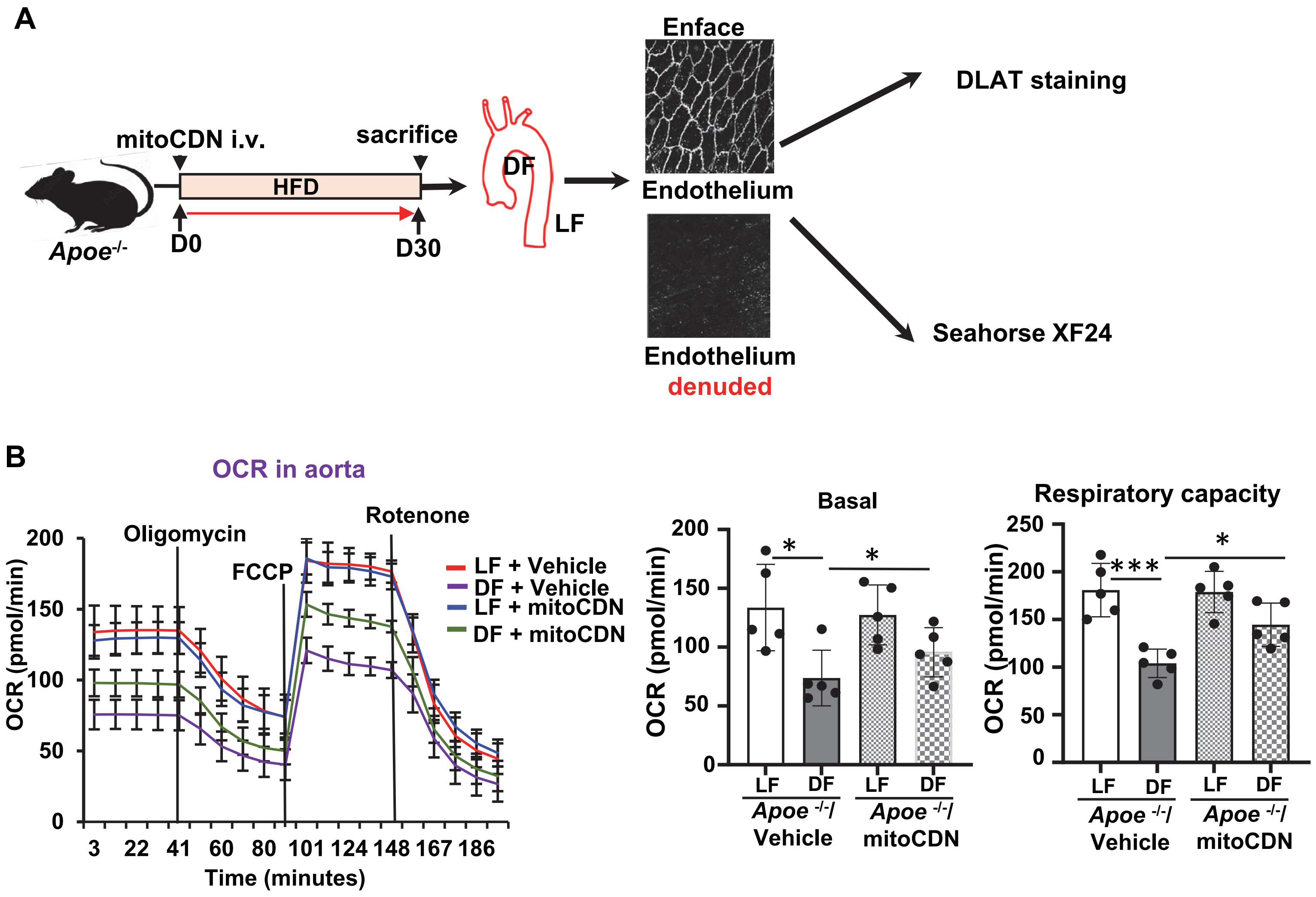

bioRxiv preprint doi: https://doi.org/10.1101/2025.01.27.634587; this version posted January 28, 2025. The copyright holder for this preprint (which was not certified by peer review) is the author/funder, who has granted bioRxiv a license to display the preprint in perpetuity. It is made available under aCC-BY 4.0 International license.

Supplementary Figure 10

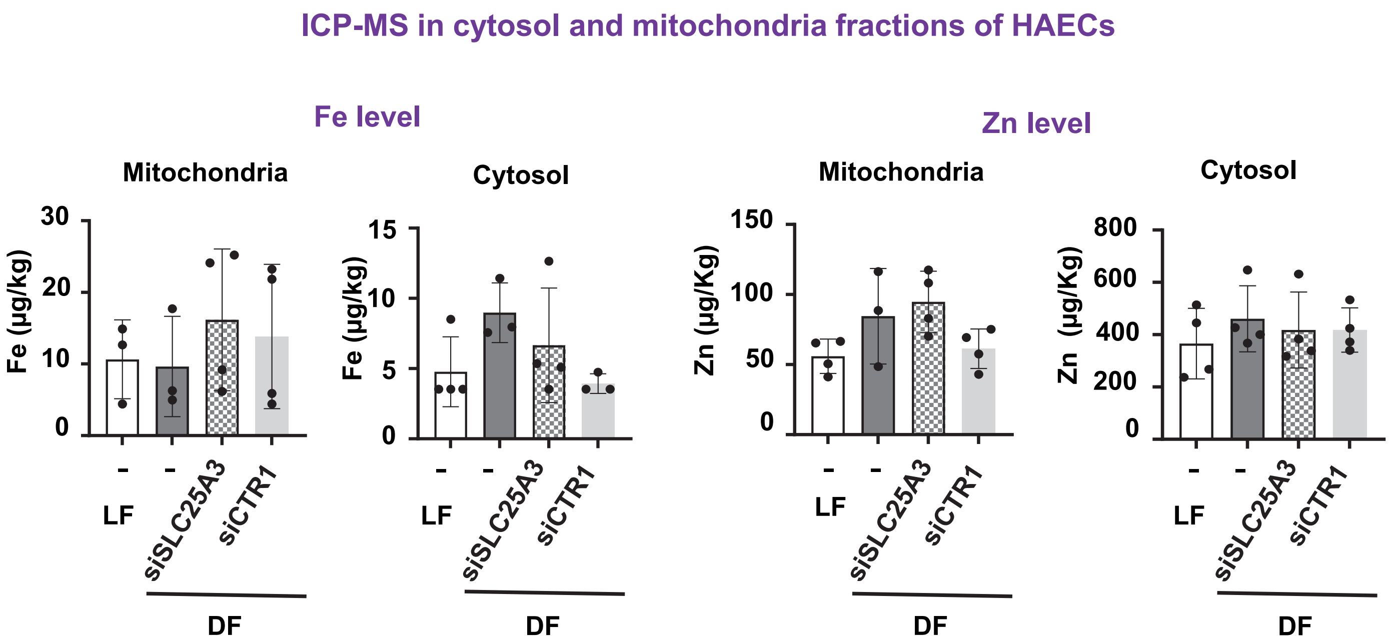

bioRxiv preprint doi: https://doi.org/10.1101/2025.01.27.634587; this version posted January 28, 2025. The copyright holder for this preprint (which was not certified by peer review) is the author/funder, who has granted bioRxiv a license to display the preprint in perpetuity. It is made available under aCC-BY 4.0 International license.

Supplementary Figure 11

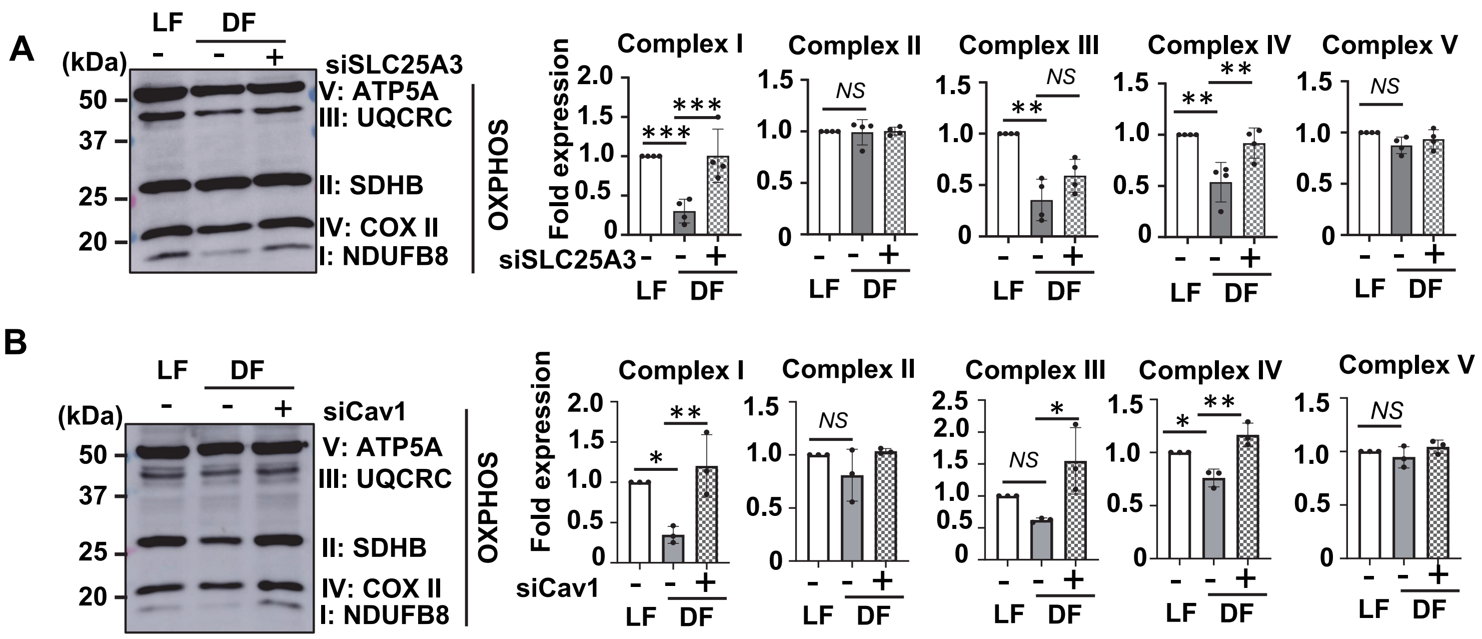

Supplement: 1 [file NIHPP2025.01.27.634587V1-supplement-1.pdf]
